# Exploring the relationships among online social capital, Internet self-efficacy, mental health, and cyberbully-victim roles in adolescents: a structural equation model

**DOI:** 10.3389/fpsyt.2025.1427655

**Published:** 2025-02-20

**Authors:** Qiqi Chen, Shaolingyun Guo, Ko Ling Chan

**Affiliations:** Department of Applied Social Sciences, The Hong Kong Polytechnic University, Hong Kong, Hong Kong SAR, China

**Keywords:** cyberbullying, online social capital, internet self-efficacy, structural equation model, adolescent

## Abstract

The prevalence and effects of cyberbullying are well-established, while there is sparse evidence addressing the experiences of those in vocational schools. Social capital and self-efficacy have attracted significant public and scholarly interest, but research on these factors in cyberspace remains limited in scope. This study aims to comprehensively investigate the pathways through which online social capital and Internet self-efficacy mediate the development of mental health consequences among adolescent cyberbullying victims. A total of 1,716 students in Grades 8-12 from public and vocational schools in China participated in the study. Structural equation modeling (SEM) was applied to specify the relationships between online social capital, Internet self-efficacy, cyberbullying, and mental health problems. Results showed that 12.12% of students reported themselves as cyber bully-victims. Internet self-efficacy could potentially mediate the effects of cyberbullying victimization and mental health problems in both school settings. Online social capital and Internet self-efficacy play mediating roles in the relationship between cyberbullying and mental health problems in public school samples. No significant effect of online social capital was found in the vocational school sample. The findings provide insights for proactive intervention in developing adequate online social capital and Internet self-efficacy training for cyberbullying prevention. Discussions on differentiated interventions for vocational school students are also presented to inspire future research and practice.

## 1 Introduction

Online interactions provide adolescents with opportunities to practice social skills and foster friendships, yet they also expose them to the growing risk of cyberbullying (1). Cyberbullying is a serious public health concern due to its association with isolation, anxiety, and stress, which can negatively impact adolescents’ overall well-being (2, 3). Unlike traditional bullying, cyberbullying creates unique challenges, as it occurs in digital spaces where anonymity, reach, and permanence amplify its effects. While much research has focused on victims and perpetrators of cyberbullying, the dual role of cyberbully-victims—individuals who simultaneously engage in cyberbullying and are themselves targets—remains underexplored. Cyberbully-victims are a particularly vulnerable group, as they navigate the compounded negative effects of both perpetrating and experiencing online abuse, including heightened guilt, shame, and social isolation (4, 5). Studies suggest that cyberbully-victims experience greater psychological distress compared to victims or perpetrators alone, including elevated risks of depressive symptoms and suicidal ideation (6). For instance, bully-victims often experience the most severe mental health outcomes due to their unique position of simultaneously causing harm and being harmed, which creates a vicious cycle of emotional turmoil (7). The prevalence of cyberbully-victims ranges between 3.3% and 24.3%, peaking during mid-adolescence, which underscores the importance of addressing this group (8). This variability reflects differences in measurement approaches and demographic factors, but it consistently underscores the need to address this subgroup within the broader context of cyberbullying research. Cyberbully-victims are more likely to have lower levels of empathy and greater impulsivity, which may contribute to their dual involvement in online aggression compared to victims or perpetrators alone (9). Furthermore, prior victimization has been shown to increase the likelihood of becoming a perpetrator, as some adolescents retaliate against their aggressors or redirect their negative emotions toward others (10). The dual role often perpetuates a cycle of harm, as these individuals may use cyberbullying as a maladaptive coping strategy to regain a sense of control or social power. However, not all individuals who experience cyberbullying will develop mental health problems (11). These findings suggest that the dual-role phenomenon is driven by complex psychosocial dynamics that differentiate cyberbully-victims from individuals who are solely victims or perpetrators. Despite the growing recognition of cyberbully-victims as a distinct group, research on this phenomenon remains limited, particularly in the context of psychosocial mechanisms that might mitigate its impact on mental health. This suggests that psychosocial mechanisms, such as online social capital and Internet self-efficacy, may mediate the relationship between cyberbullying and mental health outcomes. Understanding these mechanisms is essential to inform educational policies and intervention strategies aimed at supporting students.

Social Capital Theory offers a valuable framework for exploring the mechanisms through which online social capital and Internet self-efficacy influence cyberbullying and its outcomes (12–14) Social capital refers to the resources accumulated through social networks that facilitate individual and collective actions (15). Online social capital, an extension of traditional social capital facilitated by digital platforms, encompasses bonding social capital (close, supportive relationships) and bridging social capital (broader, informational connections) (16, 17). Adolescents build online social capital through self-presentation, self-disclosure, and participation in online communities (18, 19). However, increased online activity can also lead to heightened risk of cyberbullying victimization, particularly for individuals with pre-existing mental health vulnerabilities (20). Research has highlighted that the nature of online interactions may exacerbate or mitigate the impact of cyberbullying on mental health outcomes, making it essential to understand the role of online social capital in this context (21).

Internet self-efficacy, or the belief in one’s ability to navigate and manage online environments, can be conceptually linked to Social Capital Theory as it reflects an individual’s capacity to access, utilize, and contribute to social capital in digital spaces. Social Capital Theory emphasizes the importance of building and leveraging interpersonal networks to achieve desired outcomes, whether through emotional support, informational resources, or collective action. Internet self-efficacy aligns with this framework by enabling individuals to effectively engage with online networks, thereby fostering the development and utilization of online social capital. For example, adolescents with higher Internet self-efficacy are more likely to confidently participate in online communities, seek support, and maintain social connections, which enhances their online social capital (21–25). Conversely, lower Internet self-efficacy may hinder their ability to engage with these networks, limiting their access to the benefits of social capital and increasing their vulnerability to risks such as cyberbullying (26).

Moreover, Internet self-efficacy plays a critical role in determining how individuals navigate the complexities of online interactions, including the risks associated with cyberbullying. Adolescents with high Internet self-efficacy are better equipped to use online resources effectively, manage conflict, and seek help when needed, which can buffer against the adverse effects of cyberbullying. At the same time, the interplay between online social capital and Internet self-efficacy underscores the importance of fostering digital literacy and resilience in young people. For instance, adolescents with strong Internet self-efficacy may build online social capital more efficiently and leverage it to mitigate the negative mental health outcomes associated with cyberbullying. These dynamics highlight how Social Capital Theory provides a unifying framework for understanding both online social capital and Internet self-efficacy as key mechanisms influencing adolescents’ experiences in digital environments.

In this study, online social capital and Internet self-efficacy are conceptualized as mediators because they represent mechanisms through which cyberbullying experiences influence adolescents’ mental health outcomes. Mediation is theoretically justified when a variable explains the process or pathway through which one variable impacts another. For instance, adolescents who experience cyberbullying may suffer declines in their mental health due to disruptions in their ability to build or maintain online social capital, a key resource for emotional support and coping. Similarly, adolescents with lower Internet self-efficacy may struggle to effectively navigate online environments, limiting their ability to seek help or mitigate the adverse effects of cyberbullying. These processes align with the mediating role of psychosocial resources, as proposed by Social Capital Theory and self-efficacy frameworks (15, 22, 26). These dynamics suggest that Internet self-efficacy functions as a psychosocial pathway rather than as a conditional factor. Similarly, online social capital mediates the relationship by demonstrating how disrupted social networks and resources can amplify the psychological toll of cyberbullying.

This study emphasizes the importance of understanding cyberbullying in diverse educational contexts, particularly among vocational school students (27). Recent studies have revealed that vocational school students are more susceptible to emotional and behavioral problems than their peers in academic schools (28). This trend is consistent across various countries, where bullying and conduct problems are more prevalent among vocational school students (28). Approximately 45% of students in China transition to vocational high schools after completing nine years of compulsory education, a number that continues to grow since the 1980s (29–31). While vocational schools provide practical skills and job training in industries such as manufacturing, healthcare, and technology, these students often face social and academic disadvantages (32, 33). These challenges may influence their online interactions, making them more vulnerable to cyberbullying and its associated mental health consequences (34). Despite these risks, vocational school students remain underrepresented in cyberbullying research at both national and international levels.

Grounded in Social Capital Theory, this study aims to examine the constructs of online social capital, Internet self-efficacy, mental health, and cyberbully-victim dual roles. Understanding the differences in cyberbullying between vocational school and public school students is crucial for designing targeted prevention programs. These educational settings may present distinct social environments and access to resources, which could influence the prevalence and impact of cyberbullying. Therefore, our study aims to provide insights into the differences between vocational and public school students, informing tailored strategies to mitigate cyberbullying and support affected students. By focusing on these mechanisms, the research can provide new insights into the complex dynamics of cyberbullying, offering guidance for interventions tailored to support vulnerable adolescents. Specifically, the study examines the mediating roles of online social capital and Internet self-efficacy in the relationship between cyberbullying and mental health problems. Based on these aims, we propose three hypotheses: (H1) online social capital mediates the relationship between cyberbullying and mental health problems; (H2) Internet self-efficacy mediates the relationship between cyberbullying and mental health problems; and (H3) online social capital and Internet self-efficacy play different roles in the relationship between cyberbullying and mental health problems between vocational school and public school students.

## 2 Methods

### 2.1 Study design and procedure

The study involved students aged 13-18 in Grades 8-12 from two public middle schools in Qingdao and one vocational high school in Wuhan, China. The schools were selected using purposive sampling to enable a comparison between public and vocational school students. The inclusion criteria required participants to be enrolled in the selected schools, within the defined age group, and willing to provide informed consent. Students who were unable to complete the survey due to absence or technical issues were excluded from the study. Data collection was conducted by the research team between September and October 2022. Approximately 2,400 students from the three schools were invited to participate, and a total of 1,716 adolescents were ultimately enrolled, yielding a response rate of 71.5%. The sample consisted of 44.5% boys (n = 764), with 66.0% (n = 1,132) from public schools and 34.0% (n = 584) from vocational schools. The participants had a mean age of 14.62 years (SD = 1.70). Additional demographic details included an average of 1.04 siblings (SD = 0.89) and a mean birth order of 1.24 (SD = 0.65). The researchers obtained informed consent from both the students and their parents prior to the survey. The participants completed a web-based, self-administered questionnaire during school hours, which took approximately 30 minutes. To ensure privacy, students were seated separately in their classrooms while completing the survey. The study protocol was reviewed and approved by the Institutional Review Board of the authors’ affiliated university.

### 2.2 Measures

#### 2.2.1 Cyberbullying

The Chinese version of the European Cyberbullying Intervention Project Questionnaire (ECIPQ) was used to collect data on the participants’ experiences of cyberbullying (35, 36). The ECIPQ consists of 14 items evenly divided into two subscales measuring cyberbullying perpetration and victimization. Sample items include “I threatened someone through texts or online messages” and “I was excluded or ignored by someone on a social networking site or Internet chat room”. Participants rated the frequency of each item on a 5-point Likert scale (0 = “never”, 4 = “always”). Cyberbullying experiences were categorized into two groups: cyberbully-victim dual roles and not cyberbully-victim dual roles. “Cyberbully-victim dual roles” was based on those who reported a score of at least one on both the perpetration and victimization subscales. The overall Cronbach’s α for the ECIPQ was 0.96, with a good Cronbach’s α of 0.98 for the perpetration subscale and 0.93 for the victimization subscale in the current study.

#### 2.2.2 Online social capital

The Online Social Capital scale (17) was used to measure students’ online social capital, which consists of two subscales: online bridging social capital (7 items) and online bonding social capital (5 items). Sample questions included “Interacting with people online gives me new people to talk to” and “There is someone online I trust to help solve my problems”. Amendments were made to some items, such as rewording “If I needed an emergency loan of USD 500” to “RMB”, so that it would be suitable for use among Chinese students. Participants rated their responses on a 5-point Likert-type scale, with 1 representing strongly disagree and 5 representing strongly agree. The internal consistency reliability of this scale was α = .87 for the bridging subscale and α = .77 for the bonding subscale.

#### 2.2.3 Internet self-efficacy

The 17-item Internet Self-Efficacy scale (ISS) (26) assessed participants’ perceived confidence in completing Internet activities. The ISS consists of five factors measuring a wide range of activities related to Internet self-efficacy: Reactive (6 items), Differentiation (4 items), Organization (3 items), Communication (2 items), and Search self-efficacy (2 items). Specific sample questions in each factor include “I can be very effective using blogging sites like blogger”, “I can improve others’ well-being through the use of hyperlinks”, “I can use the Internet to answer my own questions in a productive way”, “I can use social networking sites as an effective way of connecting with others”, and “I can use the Internet to find good information about topics that are important to me”. The participants were asked to respond on a 7-point Likert scale ranging from 1 (not at all confident) to 7 (very confident). In current study, the overall Cronbach’s α for the ISS was 0.97, with good Cronbach’s α of each subscales: Reactive (0.93), Differentiation (0.92), Organization (0.93), Communication (0.86) and Search self-efficacy (0.91).

#### 2.2.4 Mental health problem

In this study, the 21-item short form of the Chinese version of the Depression Anxiety Stress Scale (DASS-21) (37, 38) was used to evaluate depression, anxiety, and stress levels of participants who were asked to reported their feelings upon cyberbullying situations. The items were categorized into three dimensions and rated on a scale of (Never), 1 (Sometimes), 2 (Often), and 3 (Always). Total scores were calculated by adding the relevant severity dimensions, which were rated on a scale of 1 (Normal), 2 (Mild), 3 (Moderate), 4 (Severe), and 5 (Extremely Severe), with a specific cut-off score assigned for each dimension. The DASS-21 demonstrated good reliability, with an overall Cronbach’s alpha of 0.93, and Cronbach’s alphas of 0.88, 0.82, and 0.90 for the Depression, Anxiety, and Stress scales, respectively.

### 2.3 Data analysis

Participants’ demographic characteristics based on cyberbullying experiences were summarized using descriptive statistics and analyzed with t-tests. Demographic factors examined included age, number of siblings, sibling rank, academic performance, and time spent online. Family-related factors, such as parental education levels and parent-child communication, were also analyzed. Mental health outcomes, including anxiety, depression, and stress, were assessed and compared between cyberbullying victims and non-victims. Additionally, gender differences in the incidence of cyberbullying were explored, but no significant differences were found.

The analysis in SEM (Structural Equation Modeling) is based on the variance and covariance of the observed variables that represent latent constructs. To ensure the validity of the SEM approach, we first assessed the normality of the data by examining skewness, kurtosis, and the distribution of all observed variables. Variables with skewness and kurtosis values within the acceptable range (± 2) were considered approximately normally distributed. Additionally, we examined multicollinearity among variables by calculating variance inflation factors (VIF), ensuring that collinearity was not an issue. These steps justified the use of Maximum Likelihood (ML) estimation, which assumes multivariate normality and is robust to minor violations of this assumption.

To handle missing data and nonresponses, we used the Expectation-Maximization (EM) algorithm, as it is an efficient iterative procedure for computing Maximum Likelihood (ML) estimates when missing values are present (39). The EM approach is particularly well-suited for SEM because it minimizes bias and improves the precision of parameter estimates. We created a correlation matrix to examine relationships among the 160 bivariate correlations between major variables and to ensure that the relationships justified inclusion in the SEM models. To address potential non-normality in the data and ensure robust parameter estimates, we used bootstrapping with 5,000 resamples. Bootstrapping is advantageous because it does not rely on strict assumptions of normality, providing bias-corrected confidence intervals for indirect effects and increasing the reliability of standard error estimates in mediation analysis.

We developed a series of SEM models to analyze the potential mediating roles of online social capital and Internet self-efficacy in the relationship between cyberbullying victimization and mental health problems. These models were based on a serial multiple mediator framework, including two types of online social capital and five types of Internet self-efficacy. Additionally, we extended the analysis to account for differences between vocational and public school students.

Multiple indices were used to assess the model fit while testing both the measurement and structural models, including Chi-square to df ratio or *χ^2^/df*, the comparative fit index (CFI) (40), Tucker–Lewis index (TLI) (41), root mean square error of approximation (RMSEA) (42), and standardized root mean square residual (SRMR) (43). The hypothesized model was tested using R software version 3.5.1 and the *Lavaan* package (44). Statistical significance was set at p <.05.

## 3 Results

### 3.1 Demographic characteristics of participants

As shown in **Table 1**, 12.12% (N = 208) reported themselves as cyberbullying victims. Older students (t = 152.82, *p <.001*), those with more siblings (t = 17.90, *p <.05*), those ranked higher among their siblings (t = 34.07, *p <.001*), and those with poorer academic performance (t = 16.01, *p <.01*) reported significantly higher scores of cyberbullying victimization. Additionally, cyberbullying victims spent more time online (t = 129.78, *p <.001*) compared to non-victims. Regarding family factors, parents of cyberbullying victims had significantly lower education levels and less communication with their children (t = 45.16*, p <.001*). Cyber bully-victims also reported significantly higher scores of anxiety (t = 178.95, *p <.01*), depression (t = 197.63, *p <.001*), and stress (t = 164.82, *p <.001*) compared to non-victims.

**Table 1:** Demographic characteristics by cyberbullying experiences.

N (%)	Total (N=1,716)	Cyber-bully-victim (N=208)	Not cyber-bully-victim (N=1,508)	Chi-square/t-test
Gender				1.21
Boy	764 (44.5)	100 (48.1)	664 (44.1)	
Girl	952 (55.5)	108 (51.9)	844 (55.9)	
Age	14.62 (1.70)	15.62 (2.36)	14.48 (1.54)	152.82^***^
Number of siblings	1.04 (0.89)	1.23 (1.15)	1.02 (0.85)	17.90^*^
Ranking in siblings	1.24 (0.65)	1.41 (0.87)	1.21 (0.61)	34.07^***^
Academic performance	2.83 (1.05)	2.79 (1.05)	3.10 (0.98)	16.01^**^
Time spent online	1.34 (1.89)	2.30 (2.15)	1.21 (1.81)	129.78^***^
Father’s education level				45.56^***^
Middle school or lower	672 (39.2)	119 (57.2)	553 (36.7)	
Higher school	559 (32.6)	60 (28.8)	499 (33.1)	
College or higher	485 (28.3)	29 (13.9)	456 (30.2)	
Mother’s education level				50.58^***^
Middle school or lower	772 (45.0)	133 (63.9)	639 (42.4)	
Higher school	523 (30.5)	48 (23.1)	475 (31.5)	
College or higher	421 (24.5)	27 (13.0)	394 (26.1)	
Communication with parents	2.52 (0.56)	2.28 (0.56)	2.55 (0.56)	45.16^***^
Anxiety	13.60 (4.86)	17.02 (5.16)	13.13 (4.63)	178.95^***^
Depression	13.05 (4.99)	16.74 (5.50)	12.54 (4.69)	197.63^***^
Stress	13.82 (5.08)	17.20 (5.30)	13.35 (4.87)	164.82^***^

*p <.05. **p <.01. ***p <.001.

### 3.2 Correlations among outcome variables

The bivariate correlations between cyberbullying victimization, online social capital, Internet self-efficacy, and mental health problems are shown in **Table 2**. We divided the correlation matrix to test the differences between school types among the variables in the SEM models. Cyberbullying victimization, all five types of Internet self-efficacy, all two types of online social capital, and all three types of mental health problems were positively correlated with each other among students in both two school types (rs ranging from 0.06 to 0.59, all *p < 0.05*). Relatively stronger correlation coefficients were found in public school students than vocational school ones. Specifically, the relationship between reactive self-efficacy and cyberbullying victimization rated the highest among the five types of Internet self-efficacy in both vocational school (r = 0.14, *p < 0.001*) and public school (r = 0.20, *p < 0.001*). The relationship between bonding social capital and cyberbullying victimization was higher than that of bridging social capital in both vocational school (r = 0.13, *p < 0.001*) and public school (r = 0.14, *p < 0.001*). The highest coefficient between cyber bullying-victimization and mental health problems was that of anxiety for vocational school (r = 0.22, *p < 0.001*) and depression for public school (r = 0.24, *p < 0.001*).

**Table 2:** Correlations among online social capital, online self-efficacy, and cyberbully-victim.

	Public Vocational	1	2	3	4	5	6	7	8	9	10	11
1	Cyberbully-victim	–	0.20^***^	0.20^***^	0.11^***^	0.09^***^	0.17^***^	0.11^***^	0.14^***^	0.24^***^	0.22^***^	0.20^***^
2	Reactive	0.14^***^	–	0.82^***^	0.76^**^	0.78^***^	0.76^***^	0.51^***^	0.59^***^	0.18^***^	0.19^***^	0.17^***^
3	Differentiation	0.08^**^	0.78^***^	–	0.79^***^	0.70^***^	0.83^***^	0.46^***^	0.53^***^	0.20^***^	0.21^***^	0.20^***^
4	Organization	0.07^**^	0.73^***^	0.85^***^	–	0.74^***^	0.83^***^	0.44^***^	0.49^***^	0.17^***^	0.17^***^	0.18^***^
5	Communication	0.09^**^	0.79^***^	0.72^***^	0.74^***^	–	0.64^***^	0.44^***^	0.49^***^	0.13^***^	0.16^***^	0.17^***^
6	Search	0.09^**^	0.74^***^	0.86^***^	0.88^***^	0.69^***^	–	0.41^***^	0.46^***^	0.22^***^	0.20^***^	0.21^***^
7	Bridging	0.11^***^	0.44^***^	0.42^***^	0.41^***^	0.40^***^	0.42^***^	–	0.82^***^	0.20^***^	0.21^***^	0.20^**^
8	Bonding	0.13^***^	0.45^***^	0.40^***^	0.38^***^	0.38^***^	0.39^***^	0.73^***^	–	0.13^***^	0.09^***^	0.11^***^
9	Depression	0.21^***^	0.14^***^	0.12^***^	0.10^***^	0.11^***^	0.09^**^	0.08^**^	0.13^***^	–	0.92^***^	0.92^***^
10	Anxiety	0.22^***^	0.19^***^	0.17^***^	0.15^***^	0.16^***^	0.15^***^	0.06^*^	0.09^**^	0.90^***^	–	0.91^***^
11	Stress	0.21^***^	0.19^***^	0.17^***^	0.15^***^	0.15^***^	0.14^***^	0.08^**^	0.11^***^	0.92^***^	0.93^***^	–

*p <.05. **p <.01. ***p <.001.

### 3.3 Vocational school model

**Figure 1:**
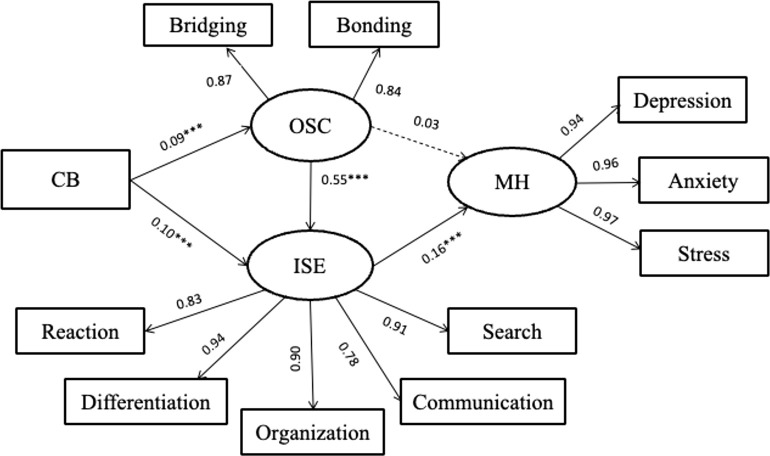
Vocational school model. *** p <.001. CB, cyber bully-victim; OSC, online social capital; ISE, internet self-efficacy; MH, mental health problems.

We then further explored and compared the above relationships between vocational and public school students. **Table 3** presented that the vocational school SEM model showed a good fit to the data (χ2 = 230.80, df = 40, CFI = 0.984, TLI = 0.976, RMSEA = 0.067, SRMR = 0.048). As shown in **Figure 1**, among students in vocational high school, cyber bullying-victimization showed a positive relationship with online social capital (βdirect = 0.09, *p <.01*) and Internet self-efficacy (βdirect = 0.10, *p <.01*). Internet self-efficacy has significant positive effect on mental health problems (βdirect = 0.16, *p <.001*). Notably, online social capital showed a positive association with Internet self-efficacy (βdirect = 0.52, *p <.001*).

**Table 3:** Model Fit statistics.

	Chi−Square	RMSEA	CFI	TLI	SRMR
Vocational school model	230.80*	0.067	0.984	0.976	0.048
Public school model	236.53*	0.093	0.968	0.970	0.046

*p <.05.

RMSEA, root mean square error of approximation; CFI, comparative fit index; TLI, Tucker–Lewis index; SRMR, standardized root mean square residual.

The bootstrapping approach was used to generate the 95% CIs (1,000 iterations) to confirm the significance of the indirect relationships in addition to the above direct effects. As shown in **Table 4**, the bootstrap results showed the predicted indirect paths to be in the expected direction, with the exception of cyber bullying-victimization through online social capital on mental health problems (βindirect = 0.050, *p > 0.05*). The indirect paths of self-efficacy on mental health problems (βindirect = 0.320, 95% CI = [0.106, 0.600]) and cyberbullying victimization through social capital to self-efficacy on mental health problems (βindirect = 0.095, 95% CI = [0.022, 0.189]) were significant.

**Table 4:** Indirect effects and estimated bootstrap CIs.

Indirect paths	Estimated Effect	95% CI
Vocational sample		
CB → OSC → MH	0.050	[-0.107, 0.344]
CB → ISE → MH	0.320^***^	[0.106, 0.600]
CB → OSC → ISE → MH	0.095^***^	[0.022, 0.189]
Public sample		
CB → OSC → MH	0.196^***^	[0.015, 0.562]
CB → ISE → MH	0.190^***^	[0.110, 0.452]
CB → OSC → ISE → MH	0.137^***^	[0.008, 0.337]

*** p <.001.

CB, cyber bully-victim; OSC, online social capital; ISE, internet self-efficacy; MH, mental health problems.

### 3.4 Public school model

**Figure 2:**
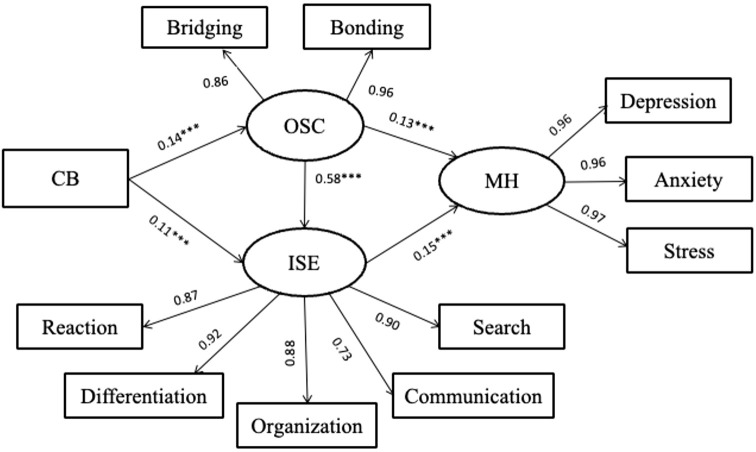
Public school model. ** p <.01, *** p <.001. CB, cyber bully-victim; OSC, online social capital; ISE, internet self-efficacy; MH, mental health problems.

**Table 3** presented that the public-school model showed a good fit to the data (χ2 = 236.53, df = 40, CFI = 0.968, TLI = 0.970, RMSEA = 0.093, SRMR = 0.046). Similarly, among public school students, the positive associations between online social capital and cyberbullying victimization (βdirect = 0.14, p <.01), and between Internet self-efficacy and cyberbullying victimization (βdirect = 0.11, *p <.01*) were observed (see **Figure 2**). In contrast to the above model, we found both online social capital (βdirect = 0.13, *p <.01*) and Internet self-efficacy (βdirect = 0.15, *p <.01*) have significant effect on mental health problems. Online social capital showed a positive relationship with Internet self-efficacy (βdirect = 0.58, *p <.001*).

The bootstrapping results indicated that the indirect paths of cyberbullying victimization through social capital (βindirect = 0.196, 95% CI = [0.015, 0.562]), through self-efficacy on mental health problems (βindirect = 0.190, 95% CI = [0.110, 0.452]) and through social capital to self-efficacy on mental health problems (βindirect = 0.137, 95% CI = [0.008, 0.337]) were significant (**Table 4**). These results demonstrated the different roles of online social capital between cyber bullying-victimization and mental health problem between public and vocational school students.

## 4 Discussion

The findings in our study demonstrated the different roles of online social capital and Internet self-efficacy among public and vocational school students in the relationship between cyberbullying victimization and mental health problems. Specifically, efforts to promote Internet self-efficacy may benefit both public and vocational school students, while enhancing online social capital may be particularly important for public school students. These findings may shed light on tailored cyberbullying programs to consider the unique mechanisms and needs of students across different educational settings.

Grounded in Social Capital Theory, our study hypothesized that online social capital mediates the relationship between cyberbullying and mental health problems. This hypothesis was confirmed for public school students but not for vocational school students. Social Capital Theory posits that social networks provide access to resources, emotional support, and opportunities for collaboration, which can buffer against stressors such as cyberbullying. For public school students, online social capital appears to serve this protective function by offering supportive networks and a sense of connection, which mitigates the negative mental health impacts of cyberbullying (12, 45). However, consistent with prior research, larger and more diverse online networks may also expose individuals to a broader range of negative social interactions, such as cyberbullying, which contributes to mental health problems (46). This dual nature of online social capital highlights its complex role in adolescents’ online experiences.

Vocational school students, on the other hand, may experience online social capital differently due to the distinct social environments in which they study. These students often focus on practical skills and job training, which may reduce their reliance on online networks for social support. Additionally, vocational students may have less cohesive or supportive online connections, limiting the buffering effects of online social capital against cyberbullying (47, 48). Furthermore, vocational schools often provide specialized interventions tailored to industry-specific challenges, which may reduce reliance on online resources for social capital (49). These findings underscore the importance of tailoring interventions to the unique needs of vocational school students by fostering supportive online networks and enhancing offline sources of social capital. In light of this difference, future research should explore these differences further to identify tailored approaches to help vocational students build supportive online networks.

We also hypothesized that Internet self-efficacy mediates the relationship between cyberbullying and mental health problems. Our findings support this hypothesis, as individuals with higher Internet self-efficacy were better able to navigate the Internet safely, reducing the impacts of cyberbullying-related stressors (26). Social Capital Theory helps explain this relationship, as individuals with higher Internet self-efficacy can more effectively use their online social networks to access resources and support, thereby improving their resilience to cyberbullying. However, higher Internet self-efficacy may also lead to greater online engagement, increasing exposure to cyberbullying and negative social interactions (12, 41). Emotion-focused coping strategies, such as avoidance, escape, anger, and depression, exacerbate mental health problems when individuals perceive cyberbullying as unchangeable (50, 51). Despite these risks, Internet self-efficacy remains a crucial skill for empowering individuals to manage online risks effectively. Further research is needed to explore how individual characteristics, such as social anxiety and existing social skills, influence the role of Internet self-efficacy in cyberbullying experiences.

We hypothesized that online social capital and Internet self-efficacy are intertwined in a way that serially mediates the relationship between cyberbullying and mental health problems. This was confirmed in both school cohorts. Adolescents with strong online social capital may feel more confident navigating the Internet and engaging in positive online interactions, enhancing their Internet self-efficacy (26). This relationship underscores the interplay between online social capital and Internet self-efficacy, as proposed by Social Capital Theory, which emphasizes the reciprocal nature of social resources and individual capacities in building resilience. To further contextualize these findings, Social Enhancement Theory and Social Compensation Theory provide additional insights. Social Enhancement Theory suggests that individuals with strong offline social capital benefit more from online interactions, as they leverage their existing networks to enhance social resources (52). In contrast, Social Compensation Theory posits that individuals with limited offline social capital rely on online interactions to expand their social networks and compensate for deficits in offline support (17). However, excessive reliance on online relationships may lead to overconfidence in Internet self-efficacy, increasing the risk of cyberbullying victimization (17). In comparison, previous studies found that higher levels of offline social capital, such as family social capital and offline emotional support were related to lower engagement in online aggression (21). Future interventions should focus on helping adolescents develop balanced online connections and fostering resilience to cyberbullying. Programs should aim to help adolescents build balanced online connections while simultaneously developing the skills and resilience needed to navigate online risks. For vocational school students, in particular, interventions may need to prioritize strengthening offline social capital to reduce over-reliance on potentially fragmented online networks.

### 4.1 Limitations

While this study uncovers important findings, it also has several limitations. Firstly, the results were gathered using self-report measures that rely on participants’ perceptions of their online social capital, Internet self-efficacy, and cyberbullying experiences. Respondents may have over-reported their social capital or under-reported their cyberbullying behaviors. It is recommended that future research employ alternative data collection methods to supplement evidence from peers or educators. Secondly, the evidence from a structural equation model limits the ability to draw definitive causal inferences from these findings. However, the study’s identification of the potential mediating roles of online social capital and Internet self-efficacy offers a foundation for future research. Alternative theoretical models, such as the Stress and Coping Framework, could also be explored in future studies to further explain these relationships. Longitudinal and experimental designs would be particularly valuable in testing causal pathways and examining how these mediators interact with different psychosocial and contextual factors over time. Thirdly, there could be other unmeasured variables that influence the relationship between vocational school and public school students, such as personal resilience, coping strategies, or family support. These factors might be more relevant in this population than the tested online social capital in cyberbullying experiences.

### 4.2 Implications

Cyberbullying can be experienced as a form of social exclusion, which can be particularly damaging to adolescents who are still developing their social identities (53). This may contribute to feelings of helplessness and isolation among those affected. The results of our study may provide implications for theoretical and practical advancement by demonstrating that both online social capital and Internet self-efficacy play complex roles in the relationship between cyberbullying and mental health problems, with a particular focus on cyberbullying victims in vocational schools. Although social capital and self-efficacy are important individual factors for child development in the digital era, studies have indicated that excessive unguided Internet use can result in negative consequences, such as Internet addiction and social alienation (54). These intricate findings emphasize the need for a more nuanced approach to comprehensively depict individualized services in cyberbullying prevention and adolescent mental health. Conceptualizing online social capital and Internet self-efficacy as mediators highlights the need for interventions that strengthen these resources to buffer against the mental health impacts of cyberbullying. For example, programs aimed at improving Internet self-efficacy could empower adolescents to better manage online risks, enhance their social capital, and access supportive networks. These interventions align with Social Capital Theory by emphasizing the importance of fostering strong and supportive digital connections. Specifically, the vocational school sample in this study may have unique characteristics, such as socio-economic background, cultural factors, or individual differences. These differences could lead to distinct online behaviors, social dynamics, and support networks, affecting the role of online social capital in mediating the relationship between cyberbullying and mental health problems (55). Unmeasured variables, such as personal resilience, family support, and coping strategies, may play a critical role in moderating the relationship between cyberbullying and mental health problems. Vocational school students, in particular, may rely on unique support systems or coping mechanisms that influence their online behavior and mental health. Incorporating these factors into future research will provide deeper insights into the psychosocial mechanisms at play and help identify additional targets for intervention.

Vocational schools, compared to public schools, often feature more targeted interventions focused on industry-specific training and support networks. However, public schools may offer broader, more general programs that better integrate social and emotional learning (48). These differences in educational structures and priorities could explain why online social capital played a less significant role in mediating cyberbullying effects among vocational students (56). Furthermore, the differentiation between online and offline social capital is becoming less distinct, as the integration of digital technologies blurs the boundaries between digital and real-life networks (57). One practical recommendation is to integrate digital citizenship education into vocational curricula. This can help students develop essential skills for responsible and ethical online behavior, including understanding the consequences of their actions, respecting privacy, and using digital tools effectively for learning and career development. Such interventions can enhance online social capital and improve Internet self-efficacy, particularly for vocational students. This study highlights the unique vulnerabilities of vocational school students, but further research is needed to examine whether the findings hold true across other educational and cultural settings. Expanding the sample to include diverse socio-economic backgrounds, geographic regions, and school types will provide a more comprehensive picture of how online social capital and Internet self-efficacy influence the relationship between cyberbullying and mental health outcomes. Additionally, future studies should investigate various forms of cyberbullying—such as harassment, doxing, and exclusion—and their differential impacts on mental health.

Individual self-efficacy levels are directly associated with previous experiences of positive reinforcement in response to the same or similar behaviors in real life (27). For mental health professionals and educators, this means that low levels of Internet self-efficacy in adolescents could result in cyberbullying being perceived as more threatening and intimidating. Similarly, teachers and mental health providers with limited Internet self-efficacy may feel ill-equipped to prevent or intervene in cyberbullying cases (58). Future programs may consider developing platforms that make tasks more engaging and intuitive, or creating educational and training strategies that offer experiences to increase individual self-efficacy in these tasks (27). This may involve providing counseling or therapy to address the emotional impact of cyberbullying, as well as education and training on responsible online behavior. Future studies may adopt longitudinal and experimental designs to establish causal relationships. Longitudinal studies tracking students over time will help clarify how cyberbullying experiences evolve and whether interventions targeting social capital and self-efficacy can produce sustained improvements in mental health. Experimental studies can test the effectiveness of specific interventions, such as digital citizenship education or self-efficacy training, in reducing the adverse effects of cyberbullying. Researchers may also investigate the interplay between online social capital and Internet self-efficacy in greater detail. This study highlights their interconnected roles in mediating the effects of cyberbullying, but future research should further distinguish between their unique contributions. For example, separating bonding and bridging online social capital may reveal distinct pathways through which these forms of social capital influence mental health outcomes. Similarly, examining how Internet self-efficacy interacts with offline social capital could uncover opportunities for integrating digital and real-world interventions. By refining our understanding of these mechanisms, future studies can inform the development of more effective, targeted interventions for adolescents at risk of cyberbullying.

## Data Availability

The raw data supporting the conclusions of this article will be made available by the authors, without undue reservation.
